# Endoscopic measurement of nasal septum perforations

**DOI:** 10.1007/s00106-021-01102-4

**Published:** 2021-10-11

**Authors:** Jean-Claude Rosenthal, Eric L. Wisotzky, Carsten Matuschek, Melanie Hobl, Anna Hilsmann, Peter Eisert, Florian C. Uecker

**Affiliations:** 1 Vision and Imaging Technologies, Fraunhofer Heinrich-Hertz-Institut HHI, Einsteinufer 37, 10587 Berlin, Germany; 2 MKG-Klinik, Charité – Universitätsmedizin Berlin, Berlin, Germany; 3 Visual Computing, Humboldt Universität zu Berlin, Berlin, Germany; 4 HNO-Klinik, Charité – Universitätsmedizin Berlin, Berlin, Germany; 5 Berlin, Germany

**Keywords:** Endoscopy, Nose diseases, Image-guided therapy, Reconstructive surgical procedures, 3D reconstruction

## Abstract

**Background:**

Nasal septum perforations (NSP) have many uncomfortable symptoms for the patient and a highly negative impact on quality of life. NSPs are closed using patient-specific implants or surgery. Implants are created either under anesthesia using silicone impressions or using 3D models from CT data. Disadvantages for patient safety are the increased risk of morbidity or radiation exposure.

**Materials and methods:**

In the context of otorhinolaryngologic surgery, we present a gentle approach to treating NSP with a new image-based, contactless, and radiation-free measurement method using a 3D endoscope. The method relies on image information only and makes use of real-time capable computer vision algorithms to compute 3D information. This endoscopic method can be repeated as often as desired in the clinical course and has already proven its accuracy and robustness for robotic-assisted surgery (RAS) and surgical microscopy. We expand our method for nasal surgery, as there are additional spatial and stereoperspective challenges.

**Results:**

After measuring 3 relevant parameters (NSP extension: axial, coronal, and NSP circumference) of 6 patients and comparing the results of 2 stereoendoscopes with CT data, it was shown that the image-based measurements can achieve comparable accuracies to CT data. One patient could be only partially evaluated because the NSP was larger than the endoscopic field of view.

**Conclusion:**

Based on the very good measurements, we outline a therapeutic procedure which should enable the production of patient-specific NSP implants based on endoscopic data only.

In ENT surgery and especially in rhinology, it is of great importance that the nasal cavity and its ventilation system are free of disturbances and are functional at the same time. A disturbance of the airflow represents a major impairment of the patient’s quality of life. This is especially true for the clinical picture of nasal septum perforation (NSP).

In NSP, breathing is impaired by the continuity defect in the cartilaginous or bony portion of the nasal septum, lacking the mucoperichondrial or mucoperiosteal lining. In Fig. 1a, the anatomical details of a wet specimen are shown in cross-section with an overview of the location of the nasal septum, while a perforation under endoscopic view is shown in Fig. 1b.

**Fig. 1:**
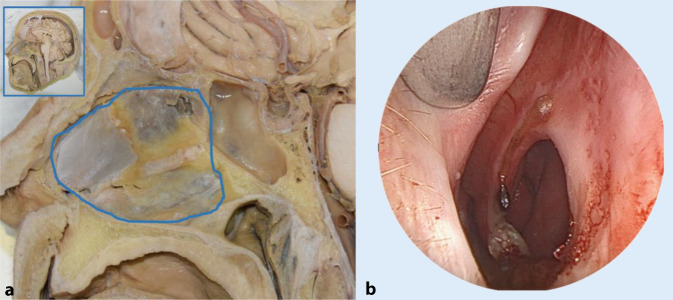
Illustration of nasal septum anatomy. **a** Wet preparation—cross section. **b** Endoscopic view of nasal septum perforation

Nasal septum perforation can lead to multiple and uncomfortable symptoms, such as restricted breathing, nosebleeds, dry or reflexively overly moist nose, and sleep disturbances. Therefore, closure of the septum is indicated to restore constant nasal airflow. Currently, there are two strategies for the treatment of NSP symptoms: (1) the use of nurturing sprays and ointments to relieve symptoms, and (2) causal surgical or nonsurgical closure of the perforation. The perforation can be temporarily closed with nasal septal implants that can be inserted or permanently surgically closed with autologous tissue. Implants are fabricated using analog and digital models. The models are generated either from silicone impressions under general or local anesthesia or from computed tomography (CT) data. However, both methods have an impact on patient safety as they increase the risk of morbidity or breach radiation hygiene.

## Objectives

This work addresses the nonsurgical treatment of NSP with temporary occlusion. For this purpose, we present a new image-based, non-contact and non-radiation measurement method using a stereo endoscope. We compare the three-dimensional (3D) endoscope measurement results with gold standard CT data. The result of this work will contribute to the extent to which endoscopic/image-based measurement methods can replace/complement established therapeutic approaches to reduce patient risk in the future.

## Clinical description of NSP

The cross-layer defect of the nasal septum (Fig. 1b) alters the velocity and volume flow in the nasal cavity. These factors are known to be critical for nasal functions such as olfaction, filtration, heating, and humidification of inhaled air [10, 26]. Posterior and smaller perforations tend to cause fewer symptoms due to the continued humidifying effect of the nasal mucosa and turbinates [2, 23]. The prevalence of a nasal septal defect ranges from 0.9% to 2.1% in the population and can be as high as 25% after septoplasty [7, 13, 15, 24]. There is no known association between septal defect and factors such as age, gender, or geographic location. The causes of NSP are diverse, and various etiologies such as traumatic perforation, occupational exposure, personal habits, substance abuse, topical or systemic medication, and certain autoimmune diseases are known [4, 8, 16, 25, 28, 32].

## Standard treatment of NSP

**Fig. 2:**
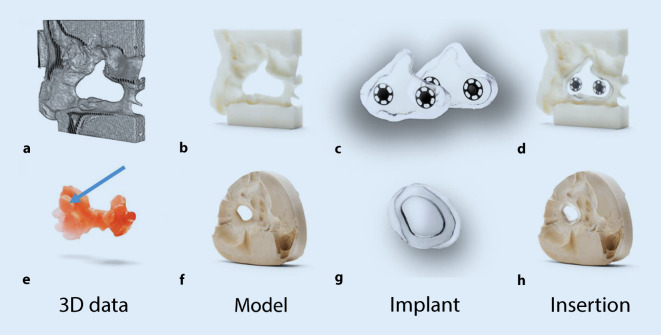
Manufacturing process for nasal septal buttons. **a–d** Computed tomography scans, **e–h** silicone impression

In principle, a nasal septal defect should be closed surgically if its size allows this [20, 21]. A restoration by means of an industrially manufactured or individually adapted button is always a compromise solution. Nonsurgical NSP therapy by temporary closure is performed with implants, so-called nasal septal buttons. These implants can be manufactured in one piece as well as in two pieces with magnets (Fig. 2c,g). A 3D model is generated from the CT data using computer-aided design (CAD) software (Fig. 2a). A physical model is then produced from the virtual data using computer-aided manufacturing (CAM) in a 3D printer (Fig. 2b). Using this printed model, the two-piece septal button with magnets is fabricated (Fig. 2c). The finished two-piece septal button must be stress-free and flush with the model (Fig. 2d). Analog impressions (Fig. 2e) provide another option for representing an NSP. A plaster model is fabricated from these impressions (Fig. 2f). Due to the size limitation of the perforation to be molded with silicone of approx. 3 cm^2^, a one-piece implant is usually fabricated for the NSP (Fig. 2g). A septal button in the analog plaster model is shown in Fig. 2h; here, likewise, the margins must be stress-free and flush. Industrially fabricated implants are almost obsolete due to their poor fit and the associated crust formation. This can lead to an enlargement of the perforation under the cofactors of local infection, manipulation by the patient or physician during cleaning, and mucosal necrosis due to inadequate care. In addition, inaccurately fitting buttons can lead to pressure points and mucosal necrosis and may result in enlargement of the perforation. The septal button is inserted through the nostril into the corresponding NSP after decongestion of the mucosa with the application of a topical local anesthetic. Two-piece septal buttons are connected to each other through the septal defect using magnets or push buttons. In general, implants are used when surgery is contraindicated for patients. In addition, therapy should begin as early as possible, since increasing perforation complicates individual treatment and leads to worsening of symptoms.

**Fig. 3:**
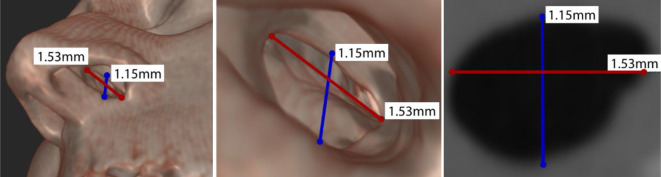
Computed tomography scan: determination of nasal septum perforation expansion in horizontal and vertical direction

However, all methods have disadvantages from the patient’s point of view: prefabricated implants are not patient-specific and have a poor fit, which can lead to crust formation. Silicone impressions are limited in size as they have reduced stability for very large defects due to lack of bearing surfaces/anchor points, they require anesthesia, and can lead to injury (increased risk of morbidity). By comparison, CT data contravene radiation hygiene. Each procedure independently requires an anatomical survey of the usually noncircular perforation. Typical perforations can range from a few submillimeters to several centimeters. When sizing, it is also important to measure the vertical as well as the horizontal length of the perforation (Fig. 3). In particular, the vertical perforation height plays a key role in the success of therapy because it has a direct impact on the tension between the floor of the nose and the bridge of the nose [4, 9]. In addition, subsequent surgical treatment is not excluded. Several techniques with different success rates have been described for surgical treatments. They are based on two basic principles: (1) the use of mucosal flaps and (2) the insertion of an interpositional element between the two mucosal surfaces [6, 14, 20–23]. However, consideration must always be made regarding the particular therapeutic method, as patients with high-grade septal deviations in particular may suffer from active bleeding. Here, we see a relative contraindication to immediate treatment in the acute bleeding situation. Furthermore, in patients with an active local infection or when drugs are to be applied intranasally, the advantages and disadvantages of the respective treatment must be critically reconsidered [4, 12, 17].

## Endoscopic 3D measurement in surgery

Image-based radiation-free 3D measurement techniques have already been used in other surgical disciplines where intraoperative support is of great interest, including robotic surgery, 3D laparoscopy, or surgical microscopy [1, 3, 11, 19, 30]. The main differences for nasal surgery are a smaller field of view and strong perspective variations due to more extreme stereoscopic viewing angles compared to visceral surgery and surgical microscopy. Considering all therapeutic, technical, and surgical conditions, there is therefore a high demand for the development of a non-contact and radiation-free measurement method for the creation of patient-specific anatomical 3D implants while increasing patient safety and implant quality.

## Methodology

**Fig. 4:**
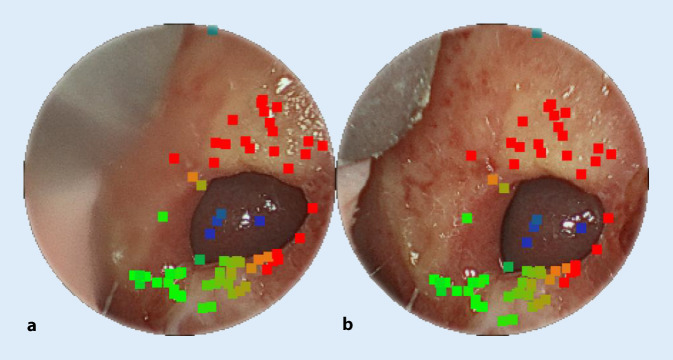
Corresponding feature points in left (**a**) and right (**b**) stereo view of nasal septum perforation for right nasal cavity

We use a stereo endoscope to capture the nasal septal defect in order to calculate a 3D digital anatomical model. For this purpose, we determine corresponding feature points that describe the same anatomical landmarks for both stereo views (see Fig. 4). From this, the so-called stereoparallax or binocular disparity can be calculated. In this manner we can perform image-based measurements using triangulation with known camera properties (including focal length) to reconstruct relevant 3D information from the endoscopic image data.

### Study group

Our method was evaluated with the agreement of six patients of different sex, age, and with varying causes of NSP (see Table 1). The study group comprised three young adults (33–36 years old), two middle-aged individuals (52–56 years old), and one older adult (81 years old) with different etiologies and duration of septal defect since the initial diagnosis.

**Table 1:** Overview of the study group

No	Sex	Age	NSP duration (months)	Reason for NSP
1	Male	36 J	38	Drug abuse
2	Male	56 J	480	Late sequelae/complication septoplasty
3	Female	35 J	52	Granulomatosis with polyangiitis/Wegener’s granulomatosis
4	Female	52 J	84	Late sequelae/complication septorhinoplasty
5	Male	81 J	264	Trauma
6	Male	33 J	26	Late sequelae/complication septoplasty

### Imaging and analysis

The stereoscopic images were acquired with two different stereo endoscopes. The first endoscope is from the company XION MEDICAL GmbH, Berlin, Germany, and was specifically designed for ENT procedures. The second endoscope is a 3D laparoscope from Schölly Fiberoptic GmbH, Denzlingen, Germany. Data from patients no. 2, 3, and 5 were acquired with the ENT endoscope (4 mm diameter, 0° optics, 80° aperture angle, 60 frames per second [fps], no zoom capability, focus changeable), while data from patients no. 1, 4, and 6 were acquired with the 3D laparoscope (10 mm, 0° optics, 72° aperture angle, 25 fps, no zoom capability, focus not changeable). Both endoscopic systems have an image resolution of 1920 × 1080 pixel per stereo channel. Our method is divided into several steps and relies on real-time image-processing algorithms [29]. First, the 3D endoscope must be calibrated since the measurement applications and stereo image processing are tightly coupled. Calibration of an optical image processing system is an offline pre-processing step that calculates the optical camera parameters such as focal length, stereo base, and lens distortions. The calibration data are valid only for a fixed setting of zoom and focus. If the magnification or the focus level is changed, this will influence the measurement accuracy. For this purpose, we use a device-independent calibration using a checkerboard-like reference body [18, 31]. Furthermore, the calibration procedure used here differs from known methods by using a model-based approach with gradient descent and image registration for correlation with the reference plane [5]. The complete 3D reconstruction and measurement chain consists of three steps: (1) stereo rectification, (2) stereoparallax/disparity estimation, and (3) metric survey of the scene. The stereo image is rectified by detecting robust feature points ([33]; Fig. 4) to derive a homography matrix that guarantees that the stereo images are free of vertical disparities. Then, disparity estimation [27] is performed using the corrected image pairs. The subpixel accurate disparity estimation considers temporal–spatial dependencies between correspondence points to be determined. In this process, the correspondences are determined locally. The iterative and independent distribution of the correspondences guarantees that the entire scene is globally updated while maintaining temporal–spatial consistency. After a short initialization phase of 20 stereo image pairs, we obtain a complete representation of the scene as a disparity map. Finally, we reconstruct the NSP anatomy to scale from the computed disparity maps and the predetermined camera calibration data. On the reconstructed endoscopy data, we then measure the horizontal and vertical dimensions using point-to-point measurements as well as the perimeter of the perforation directly in the image. The perimeter is calculated by accumulating multiple point-to-point measurements. For the comparison, there was no registration of the measurement points to the CT data. But at the same time, the comparison measurements between endoscopy images and CT data were performed on the same anatomical sites.

## Results

**Fig. 5:**
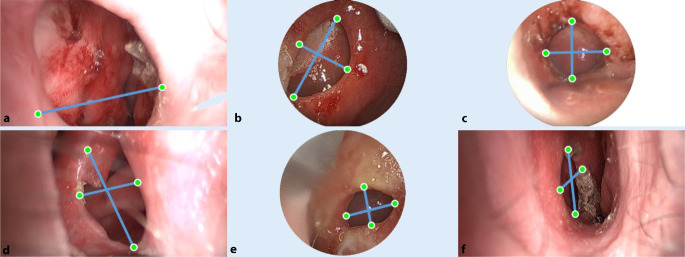
Overview of the nasal septum perforations of the study group from Table 1 and visualization of the axial and coronal measurement points. The corresponding measurement results are shown in Table 2. **a **Patient 1,** b **patient 2**, c **patient 3**, d **patient 4**, e **patient 5**, f **patient 6

The perforations of all six patients were imaged with one of the endoscopes described. The existing perforations are shown in Fig. 5 with selected measurement points. Table 2 gives an overview of the image-based measurement results and compares them with the existing CT measurements. The slice thickness of the CT data is 0.5 mm for patient 1 and 0.625 mm for all the rest. The corresponding DICOM data were analyzed with the ImFusion Suite software (ImFusion GmbH, Munich, Germany). For all patients except patient 1, the axial and coronal axes as well as the extent of perforation were determined. The relative deviations were within the required measurement accuracy of 5%. The mean of all absolute differences is 0.28 mm with a standard deviation (SD) of 0.146 mm for the axial axis and 0.16 mm with an SD of 0.09 mm for the coronal axis, and 0.904 mm with an SD of 0.769 mm for the circumference. For all measurements performed, the absolute errors were less than 0.5 mm and thus can provide comparable measurements to CT data. There are no significant differences between the two endoscopic systems in terms of accuracy and measurement uncertainty. The measurement accuracy in this case is determined by the parameters of the stereo system: focal length, stereo base, image resolution, pixel size, as well as the most distant point included in the measurement. Patient no. 1 requires further explanation (Fig. 5a), as the proposed method did not allow a meaningful axial measurement to be acquired and therefore no determination of the circumference. There are two main reasons for this. First, it was not possible to record the perforation extent with a single image because the endoscopic field of view was exceeded in the axial dimension due to the extreme size of the perforation of 4.50 cm.

**Table 2:** Evaluation and comparison of measurement results for stereo endoscope and computed tomography (CT) data

	Stereo-endoscopic measurement (mm)				CT measurement (mm)				Relative measurement deviation (%)		
No.	Axial	Coronal	Circumference	SD	Axial	Coronal	Circumference	Voxel size	Axial	Coronal	Circumference
1	–	18.8	–	0.3	45.0	19.1	12.30	0.6	–	1.57	–
2	9.60	7.30	27.96	0.2	9.20	7.40	27.30	0.6	4.35	1.35	2.42
3	15.5	11.8	44.39	0.2	15.3	11.5	44.70	0.6	1.31	2.61	0.69
4	11.3	15.1	43.50	0.2	11.1	15.0	42.30	0.6	1.80	0.67	2.84
5	7.40	5.60	20.20	0.4	7.50	5.40	20.30	0.6	1.33	3.70	0.49
6	24.4	9.9	63.05	0.7	24.9	10.0	60.80	0.6	2.01	1.00	3.70

Furthermore, important areas could not be calculated due to stereoscopic occlusion caused by an acute viewing angle. Second, image quality was negatively affected by camera motion blur and by condensation on the endoscope optics due to breathing. Motion blur occurs on both lenses, while condensation can also occur on only one lens. In both cases, the image quality decreases sharply and it is no longer possible to find reliable correspondences from which to calculate the required depth information.

**Fig. 6:**
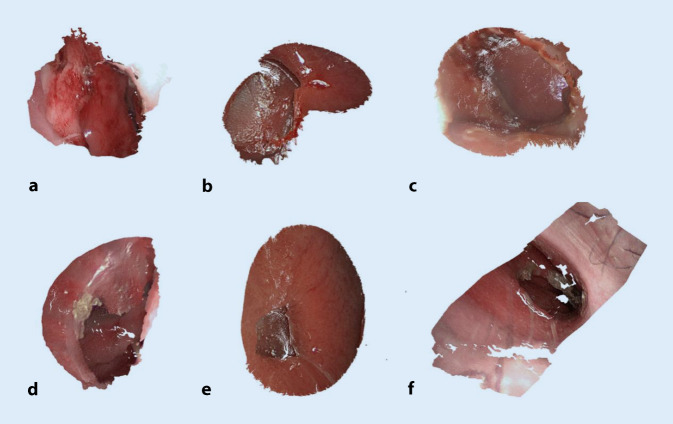
Overview of patient anatomies with nasal septum perforations as textured 3D point clouds. **a **Patient 1,** b **patient 2**, c **patient 3**, d **patient 4**, e **patient 5**, f **patient 6

In addition to the purely image-based relevant survey parameters, the associated 3D point clouds (see Fig. 6) were also fully computed to enable targeted 3D visualization for quantitative assessment of the anatomy. The image-based 3D reconstruction method used here has already been evaluated in robotic-assisted surgery on 3D reference anatomies of porcine cadavers. It is real-time capable and provides very accurate measurement results with very low error rates in the target/actual comparison to the known reference variable [1]. Thus, the results obtained here confirm the general performance of the 3D reconstruction method for another surgical domain, with additional challenges for the algorithms resulting from the complex nasal anatomy as well as the difficult stereo-perspectives viewing angles.

## Discussion and outlook

We have presented a new image-based approach for measuring key anatomy parameters in NSP using a 3D endoscope and compared it with CT data. Our method shows accurate and precise measurement results with small relative deviations in the submillimeter range for determining the true-to-scale dimension of the NSP in the horizontal and vertical directions, as well as determining the perforation circumference. With the image-based method, the mucosal defect can be precisely detected, but in contrast to the CT-based method, there is no optical detection of the cartilage defect, which can be significantly larger than the mucosal defect. Currently, the measurements are still performed with a 2D image. Graphical CT measurements continue to have the advantage that present septal deviations as well as individually adapted buttons can be better recorded or integrated. This also applies to the recording of the thickness of the septum at the anterior and posterior perforation margin. Furthermore, it is already possible to generate high-resolution 3D point clouds, which allow for a quantitative as well as qualitative assessment of the patient’s anatomy by surgeons and, in addition, allow one to capture the aforementioned challenges in comparison with CT measurements.

The next steps will be to investigate the practical feasibility of this approach, which is gentle for the patient, in order to replace the two current nonsurgical methods (CT scan, silicone impression) for the treatment of NSP. Among other things, the procedure needs to be extended so that the crucial NSP parameters can be measured for all perforation sizes. This is especially true for nasal septal defects larger than approximately 3 cm^2^, where the surgical scene cannot be captured with a single image. The treatment of large septal defects can be approached promisingly with the evidence provided here of highly accurate measurement results from single images. For this purpose, the measurements and the surface reconstruction for both nostrils will be registered and fused to each other with so-called 3D mosaic and SLAM (simultaneous localization and mapping) methods to form a global 3D model, so that this can be used for 3D printing of the implant and easily integrated into the existing workflows.

## Practical conclusion

- Intraoperative image-based measurement procedures with three-dimensional endoscopes achieve comparable measurement values to computed tomography (CT) data while avoiding radiation exposure and can be repeated as often as required in the clinical course.
- Endoscopes are inexpensive sensors compared to CT devices with the possibility to specifically extend their functionality by additional artificial intelligence algorithms.
- Three-dimensional reconstruction from endoscopic image data forms the basis for future augmented reality applications in all surgical disciplines.
- Three-dimensional reconstruction methods form the general basis for patient-specific implant solutions in ENT surgery.

## Declarations

All research involving human subjects complies with all relevant national regulations, institutional guidelines and has been conducted in accordance with the principles of the Declaration of Helsinki and approved by the Ethics Committee Charité - Universitätsmedizin Berlin.

The supplement containing this article is not sponsored by industry.

## References

[1] Allan M, Mcleod J, Wang CC, Rosenthal JC, Fu KX, Zeffiro T, Xia W, Zhanshi Z, Luo H, Jia F, Zhang X, Li X, Sharan L, Kurmann T, Schmid S, Psychogyios D, Azizian M, Stoyanov D, Maier-Hein L, Speidel S (2021) Stereo correspondence and reconstruction of endoscopic data challenge https://arxiv.org/abs/2101.01133

[2] Bhattacharyya N (2007). Clinical symptomatology and paranasal sinus involvement with nasal septal perforation. Laryngoscope.

[3] Bodenstedt S, Wagner M, Mayer B, Stemmer K, Kenngott H, Müller-Stich BP, Dillmann R, Speidel S (2016). Image-based laparoscopic bowel measurement. Int J Comput Assist Radiol Surg.

[4] Downs BW, Sauder HM (2020). Septal perforation.

[5] Eisert P (2002). Model-based camera calibration using analysis by synthesis techniques.

[6] de Gabory L, Bareille R, Stoll D, Bordenave L, Fricain J-C (2010). Biphasic calcium phosphate to repair nasal septum: the first in vitro and in vivo study. Acta Biomater.

[7] Gold M, Boyack I, Caputo N, Pearlman A (2017). Imaging prevalence of nasal septal perforation in an urban population. Clin Imaging.

[8] Kridel RWH (1999). Septal perforation repair. Otolaryngol Clin North Am.

[9] Lee JY, Lee SH, Kim SC, Koh YW, Lee SW (2006). Usefulness of autologous cartilage and fibrin glue for the prevention of septal perforation during septal surgery: a preliminary report. Laryngoscope.

[10] Lindemann J, Keck T, Wiesmiller K, Sander B, Brambs H-J, Rettinger G, Pless D (2006). Nasal air temperature and airflow during respiration in numerical simulation based on multislice computed tomography scan. Am J Rhinol.

[11] Maier-Hein L, Groch A, Bartoli A, Bodenstedt S, Boissonnat G, Chang PL, Clancy NT, Elson DS, Haase S, Heim E, Hornegger J, Jannin P, Kenngott H, Kilgus T, Müller-Stich B, Oladokun D, Röhl S, dos Santos TR, Schlemmer HP, Seitel A, Speidel S, Wagner M, Stoyanov D (2014). Comparative validation of single-shot optical techniques for laparoscopic 3-D surface reconstruction. IEEE Trans Med Imaging.

[12] Neumann A, Schneider M, Tholen C, Minovi A (2010). Inoperable Nasenseptumdefekte. HNO.

[13] Oberg D, Åkerlund A, Johansson L, Bende M (2003). Prevalence of nasal septal perforation: the Skövde population-based study. Rhinology.

[14] Parry JR, Minton TJ, Suryadevara AC, Halliday D (2008). The use of fibrin glue for fixation of acellular human dermal allograft in septal perforation repair. Am J Otolaryngol.

[15] Peacock MR (1981). Sub-mucous resection of the nasal septum. J Laryngol Otol.

[16] Power DG, Kemeny NE (2011). Nasal septum perforation and bevacizumab. Med Oncol.

[17] Romo T, Sclafani AP, Falk AN, Toffel PH (1999). A graduated approach to the repair of nasal septal perforations. Plast Reconstr Surg.

[18] Rosenthal JC, Gard N, Eisert P (2017). Kalibrierung stereoskopischer Systeme für medizinische Messaufgaben.

[19] Rosenthal J-C, Gard N, Schneider A, Eisert P (2018). Microscopic image-based determination of stapes prosthesis length.

[20] Scheithauer M, Lindemann J, Sommer F, Wigand MCC (2021). Closure of nasal septal perforation. Laryngorhinootologie.

[21] Schultz-Coulon H-J (2005). Three-layer repair of nasoseptal defects. Otolaryngol Head Neck Surg.

[22] Susman E (2007) Fibrin glue makes septal perforations easier to repair. https://www.enttoday.org/article/fibrin-glue-makes-septal-perforations-easier-to-repair/. Zugegriffen: 24. Febr. 2020

[23] Tasca I, Compadretti GC (2006). Closure of nasal septal perforation via endonasal approach. Otolaryngol Head Neck Surg.

[24] Topal O, Celik SB, Erbek S, Erbek SS (2011). Risk of nasal septal perforation following septoplasty in patients with allergic rhinitis. Eur Arch Otorhinolaryngol.

[25] Traina TA, Norton L, Drucker K, Singh B (2006). Nasal septum perforation in a bevacizumab-treated patient with metastatic breast cancer. The Oncol.

[26] Uecker FC (2013). Hals-Nasen-Ohren-Heilkunde in Frage und Antwort.

[27] Waizenegger W, Feldmann I, Schreer O, Kauff P, Eisert P (2016). Real-time 3D body reconstruction for immersive TV.

[28] Watson D, Barkdull G (2009). Surgical management of the septal perforation. Otolaryngol Clin North Am.

[29] Wisotzky EL, Rosenthal J-C, Eisert P, Hilsmann A, Schmid F, Bauer M, Schneider A, Uecker FC (2019). Interactive and multimodal-based augmented reality for remote assistance using a digital surgical microscope.

[30] Wisotzky EL, Rosenthal J-C, Wege U, Hilsmann A, Eisert P, Uecker FC (2020). Surgical guidance for removal of cholesteatoma using a multispectral 3D-endoscope. Sensors.

[31] Zhang Z (1999). Flexible camera calibration by viewing a plane from unknown orientations.

[32] Zhao K, Dalton P (2007). The way the wind blows: implications of modeling nasal airflow. Curr Allergy Asthma Rep.

[33] Zilly F, Riechert C, Eisert P, Kauff P (2011). Semantic kernels binarized-a feature descriptor for fast and robust matching.

